# Genomic Insights of *Dyadobacter tibetensis* Y620-1 Isolated from Ice Core Reveal Genomic Features for Succession in Glacier Environment

**DOI:** 10.3390/microorganisms7070211

**Published:** 2019-07-22

**Authors:** Liang Shen, Yongqin Liu, Ninglian Wang, Namita Paudel Adhikari

**Affiliations:** 1 Key Laboratory of Tibetan Environment Changes and Land Surface Processes, Institute of Tibetan Plateau Research, Chinese Academy of Sciences, Beijing 100085, China; 2 CAS Center for Excellence in Tibetan Plateau Earth Sciences, Chinese Academy of Sciences, Beijing 100085, China; 3 College of Life Sciences, Anhui Normal University, Wuhu 241000, China; 4 College of Resources and Environment, University of Chinese Academy of Sciences, Beijing 100190, China; 5 College of Urban and Environmental Science, Northwest University, Xian 710069, China

**Keywords:** glacier, ice core, genomics, *Dyadobacter*

## Abstract

Glaciers have been recognized as biomes, dominated by microbial life. Many novel species have been isolated from glacier ecosystems, and their physiological features are well characterized. However, genomic features of bacteria isolated from the deep ice core are poorly understood. In this study, we performed a comparative genomic analysis to uncover the genomic features of strain *Dyadobacter tibetensis* Y620-1 isolated from a 59 m depth of the ice core drilled from a Tibetan Plateau glacier. Strain *D. tibetensis* Y620-1 had the smallest genome among the 12 cultured *Dyadobacter* strains, relatively low GC content, and was placed at the root position of the phylogenomic tree. The gene family based on a nonmetric multidimensional scaling (NMDS) plot revealed a clear separation of strain *D. tibetensis* Y620-1 from the reference strains. The genome of the deep ice core isolated strain contained the highest percentage of new genes. The definitive difference is that all genes required for the serine-glyoxylate cycle in one-carbon metabolism were only found in strain *D. tibetensis* Y620-1, but not in any of the reference strains. The placement of strain *D. tibetensis* Y620-1 in the root of the phylogenomic tree suggests that these new genes and functions are of ancient origin. All of these genomic features may contribute to the survival of *D. tibetensis* Y620-1 in the glacier.

## 1. Introduction

Glaciers and ice sheets comprise approximately 70% of the total freshwater on Earth [1]. Although they are the largest freshwater reservoirs on Earth, only recently have those systems been recognized as biomes dominated by microorganisms [1,2,3]. Microbe-mediated biogeochemical cycles on glaciers have both local and global impacts [2,4]. Thus, it is important to understand the physiology and genomic features of these microorganisms.

In spite of the fact that the glacial environment is too hostile for the proliferation and survival of advanced organisms, the snow and ice ecosystems are not so extreme for microorganisms [5,6], and viable microorganisms have been found in ice cores drilled from polar and Tibetan Plateau glaciers [7,8]. Interconnected liquid veins along three-grain boundaries in ice were proposed as a habitat in which psychrophilic bacteria can move and obtain energy and carbon from the solution in the liquid veins [8]. Recently, many novel species have been described from glaciers in the Alps [9,10,11], Tibetan Plateau [12,13,14,15,16], Antarctic [17,18], and Arctic [19,20,21], suggesting that the cold origin of endemic species [22]. To survive in cold environments, psychrophilic bacteria possess special adaptation strategies in terms of both physiology and molecular basis [23,24,25]. The physiological features (e.g., growth temperature, salinity, pH; composition of fatty acids, menaquinone; enzyme activities and assimilation of general carbon sources) have been well described. However, the genomic features of these glacier isolated type strains are poorly characterized.

In the present study, the genome of a type strain *Dyadobacter tibetensis* Y620-1 isolated from a 59 m depth of the ice core drilled from Yuzhufeng Glacier on the Tibetan Plateau was compared to the genomes of 12 *Dyadobacter* cultured isolates, and one metagenome assembled genome. The genus *Dyadobacter* was first proposed by Chelius and Triplett [26], within the phylum Bacteroidetes, class Sphingobacteria. Bacterial members of this genus are gram-negative rods that have been isolated from diverse habitats, i.e., plant, soil, freshwater, seawater, glacier, subterranean sediment, and desert sand [27]. Our aim was to investigate the genomic features of the deep ice core isolated strain *D. tibetensis* Y620-1 and identify the potential strain specific metabolism pathways that facilitate its survival in the glacial environments.

## 2. Materials and Methods

Ice core samples were drilled from the Yuzhufeng Glacier on the Tibetan Plateau of China (94° 14.77′ E, 35° 39.64′ N) in 2009. The type strain Y620-1 was isolated from a 59 m depth of the ice and has been proposed as a novel species named as *Dyadobacter tibetensis* Y620-1 [28].

The genome of strain *D. tibetensis* Y620-1 was sequenced in 2012 and described by Liu et al. (2014). The reference genomes were downloaded from the NCBI database in March 2018 (Table 1). The completeness of genomes was estimated using CheckM [29], genomes with a completeness of less than 95% and contamination over 5% were removed. AAI and ANI (Average nucleotide and amino acid identity) were calculated using compareM: https://github.com/dparks1134/CompareM [30] and ANI calculator http://enve-omics.ce.gatech.edu/ani/ [31], respectively. To remove potential differences introduced through different annotation methods, all the genomes analyzed were annotated simultaneously in the present study with RAST (Rapid Annotation using Subsystem Technology) [32] and PROKKA [33].

For phylogenomic clustering, *Runella limosa* DSM 17973 and *Rudanella lutea* DSM 19387 were chosen as the out-group. The two strains are close relatives to the *Dyadobacter* genus [34] and are placed right at the lineage outside *Dyadobacter*. In general, out-groups that are closely related to the in-group species are better suited for phylogeny reconstruction than the distantly related ones [35]. The Maximum Likelihood phylogenomic tree was constructed using PhyloPhlAn2 [36]. Neighbor-Joining and Bayesian trees were constructed using MEGA 5.05 and Mrbayes 3.2, respectively, with the concatenated protein sequences produced by PhyloPhlAn2 [37,38,39].

Gene families were clustered using FastOrtho software (--pv_cutoff 1-e5 --pi_cutoff 70 --pmatch_cutoff 70): http://enews.patricbrc.org/fastortho/ [40] the cutoff values were set according to Parks et al. (2017). Gene family matrix was produced using custom-made PERL scripts. Ordinations and statistical analyses were performed using the vegan package v2.4.4 [41] using R v3.3.3.

## 3. Results

### 3.1. General Features of the Dyadobacter Genomes

*Dyadobacter* strains with high quality non-redundant genomes were isolated from a wide range of habitats, e.g., soil, desert sand, fresh water, plant, and bioreactor (Table 1). The size of the *Dyadobacter* genomes ranged from 5.18 to 8.74 Mbp. Out of the 13 *Dyadobacter* genomes, 12 were cultured with completeness >99.69 %, and one (*Dyadobacter* sp. UBA7685) was assembled from the metagenome of a water sample with completeness of 97.02%. The genome size of the strain *D. tibetensis Y620-1* (5.31 Mb) was the smallest among the 12 cultured *Dyadobacter* strains. The genomic GC content (guanine-cytosine content) of the 13 *Dyadobacter* genomes ranged from 41.26% to 52.08%. Most of the strains that were able to grow at ≤5 ℃ have considerably lower GC contents (≤47.00 %) than those with a minimum growth temperature ≥10 ℃ (GC content ≥50.23 %) [16,26,42,43,44,45,46,47,48]. The genomic GC content of strain *D. tibetensis* Y620-1 was 43.45%, which was lower than all the *Dyadobacter* genomes, except for the strain *D. koreensis* DSM 19938 (41.26 %). The CRISPRs (Clustered regularly interspaced short palindromic repeats) were only identified from strain *D. tibetensis* Y620-1 and *Dyadobacter* sp. 50-39 with 6 and 5 copies, respectively. Seven strains were predicted to contain a full rRNA operon. The copy number of 16S rRNA varied widely (from 1 to 4 copies) in the genomes of *Dyadobacter*. For example, the genome of strain *D. tibetensis* Y620-1 contained one 16S rRNA gene, while strain *D. fermentans* DSM 18053 had four copies of 16S rRNA genes. Harboring a lower copy number of rRNA operon suggested strain *D. tibetensis* Y620-1 being having an oligotrophic lifestyle [49]. The copy number of tRNA ranged from 30 to 43 in the *Dyadobacter* genomes. The 13 genomes contained 3 to 7 copies of cold shock genes. Strain *D. tibetensis* Y620-1 contained the largest number of cold shock genes among the 13 genomes with 5 *CspA* and 2 *CspG* genes been identified. Other components of csp family (*CspB*, *CspC*, *CspD*, *CspE*, *CspF* and *CspI*) were not contained by any of the 13 genomes.

### 3.2. Distribution of Dyadobacter Strains in Their Phylogenomic Tree

**Figure 1:**
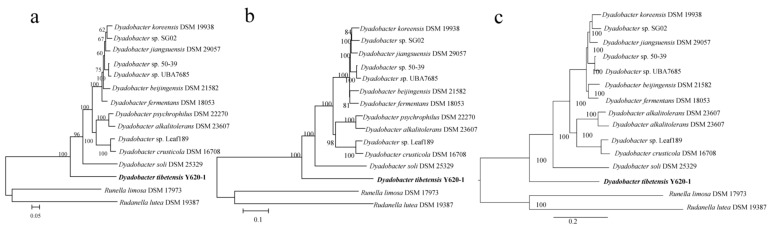
(**a**) Phylogenomic clustering of *Dyadobacter* strains based on concatenated alignment orthologous proteins using PhyloPhlAn; (**b**) Neighbor-Joining tree constructed by MEGA; (**c**) Bayesian tree constructed by Mrbayes. Numbers at nodes indicate bootstrap percentages for ML (Maximum Likelihood) and NJ (Neighbor Joining) tree, and posterior probabilities for Bayesian tree. Bar 0.05, 0.1 and 0.2 accumulated changes per amino acid for ML, NJ and Bayesian tree, respectively.

To infer the ancestral state, a robust phylogenomic tree is needed to describe the evolutionary relationship of the taxa. We obtained a robust evolutionary position of the 13 Dyadobacter strains using three different phylogenomic approaches (ML (Maximum Likelihood) and NJ (Neighbor Joining), and Bayesian, Figure 1). Strains isolated from different environments were mixed in the phylogenomic tree. Strain *D. koreensis* DSM 19938 and *Dyadobacter* sp. UBA7685 isolated from fresh water were located in the deep lineage with strains isolated from the soil and bioreactor. The plant associated strain *Dyadobacter* sp. Leaf189 was placed in the middle lineage with strains isolated from the soil and desert sand. Strain *D. tibetensis* Y620-1 was isolated from the 59 m depth of an ice core, with the smallest genome placed in the basal position of the phylogenomic tree.

### 3.3. Average Nucleotide and Amino Acid Identity

**Figure 2:**
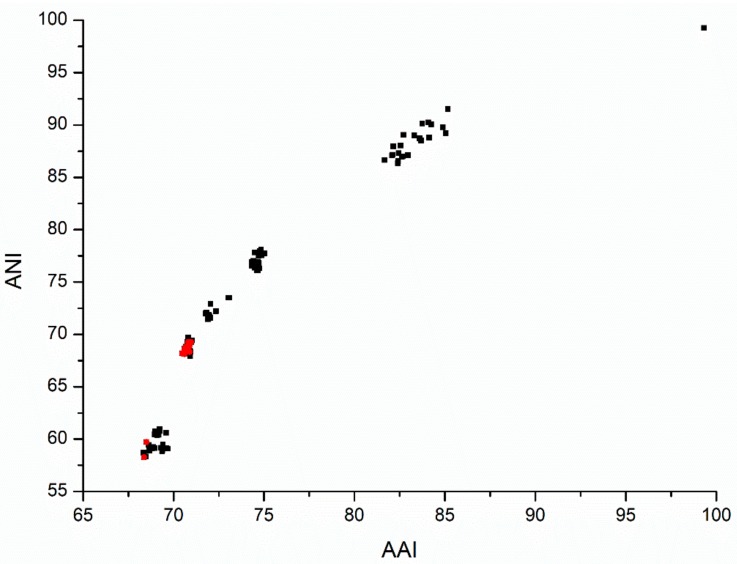
Relationships between average nucleotide (ANI) and amino acid identity (AAI), black dots for all pairs of the genomes and red dots for *D. tibetensis* Y620-1 and the reference genomes.

We calculated the pairwise AAI and ANI of the *Dyadobacter* with the two out-group strains. The inter-genus AAI and ANI were not higher than 69.70% and 60.91%, respectively. The intra-genus ANI and AAI ranged from 70.48% to 99.33% and 67.92% to 99.29%, respectively (Figure 2). The highest pairwise AAI values observed was 99.33% between strain *Dyadobacter* sp. UBA7685 assembled from metagenome and *Dyadobacter* sp. 50-39 isolated from a bioreactor, suggesting that all investigated genomes represented non-redundant genomes based on the proposed threshold of 99.5% AAI suggested by Parks et al. (2017). The rest of the pairwise ANI were all lower than 95%, suggesting that these are different species [50]. Thus, the 13 *Dyadobacter* genomes could represent 12 distinct species (*Dyadobacter* sp. UBA7685 and *Dyadobacter* sp. 50-39 could be the same species). Genome clustering based on AAI and ANI matrix was consistent with their phylogenomic positions, for example, *Dyadobacter* sp. UBA7685 and *Dyadobacter* sp. 50-39 with the highest ANI were placed together (Figure 1 and Figure 2).

### 3.4. Distribution Pattern of Function Genes and Gene Families

We annotated the *Dyadobacter* genomes on the RAST server. The functional genes were classified into four hierarchy levels: category, subcategory, subsystem and role. There were 26 categories, 99 subcategories, 372 subsystems and 1498 roles identified, and no substantial differences were observed among the 13 genomes (Table S1). The distribution of genes in the 26 categories did not differ significantly between strain *D. tibetensis* Y620-1 and the reference strains (chi-square test, *P* > 0.05). At the subcategory level, nine genes related to inorganic sulfur assimilation were specific to strain *D. koreensis* DSM 19938 and were not identified in other genomes. There were 16 strain specific subsystems in the 13 *Dyadobacter* genomes. Most interestingly, twenty-five genes related to serine-glyoxylate cycle were specific to strain *D. tibetensis* Y620-1, and seventeen genes related to L-fucose utilization were specific to strain *Dyadobacter* sp. SG02. There were 134 specific roles distributed in 12 *Dyadobacter* genomes except *Dyadobacter* sp. UBA7685. Strain *D. koreensis* DSM 19938 and *D. tibetensis* Y620-1 were very divergent with 48 and 33 strain specific roles, and the rest had no more than 20.

**Figure 3:**
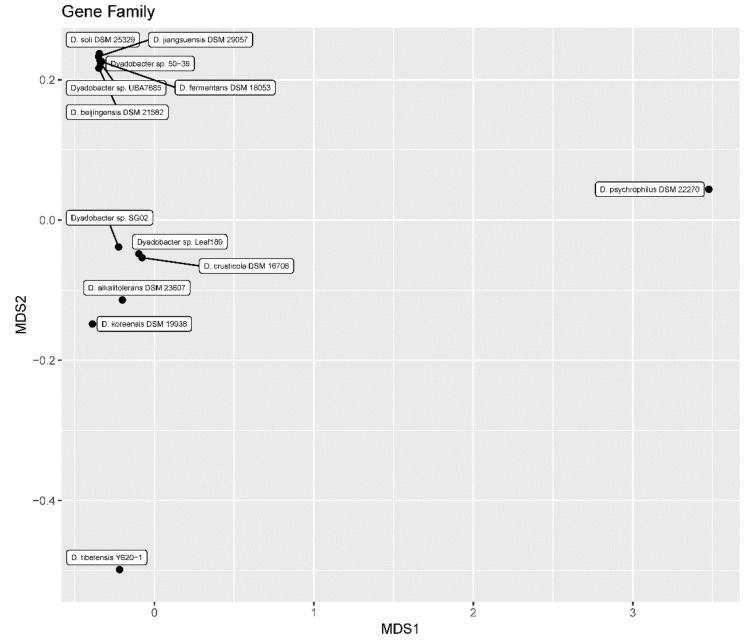
Nonmetric multidimensional scaling (NMDS) plot of gene family showing clear separation of the two cold adapted strains *D. tibetensis* Y620-1 and *D. psychrophilus* DSM 22270.

We constructed a gene family matrix of the 13 *Dyadobacter* genomes. Genes in these genomes were clustered into 10,898 families, alternatively pan genomes. The Core genome of the 13 *Dyadobacter* genomes comprised 1382 gene families (Table S2). The number of gene families (10,898) was much higher than the function type of the genes (1498 types, defined by RAST), suggesting a high sequence diversity of genes with the same function in the *Dyadobacter*. Ordination of functional genes using two-dimensional non-metric multidimensional scaling (NMDS) revealed a clear separation of strain *D. tibetensis* Y620-1 and *D. psychrophilus* DSM 22270 (Figure 3). Strain *D. psychrophilus* DSM 22270 was isolated from hydrocarbon contaminated soil, and it is a psychrophilic bacterium [44].

**Figure 4:**
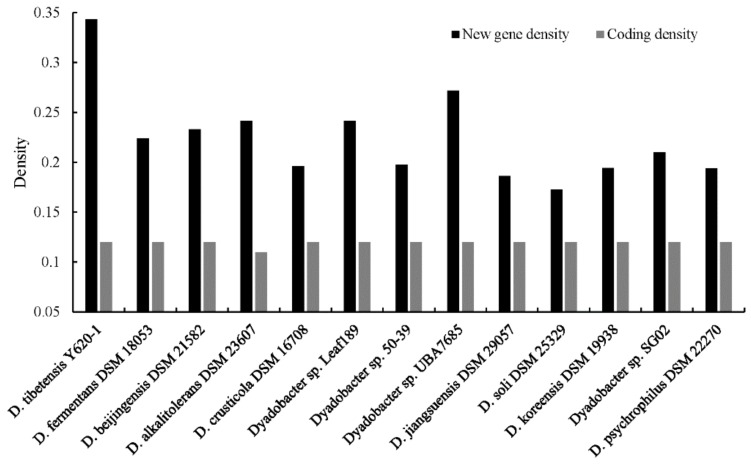
Bar graph showing the density of new genes and protein coding genes in each genome.

**Figure 5:**
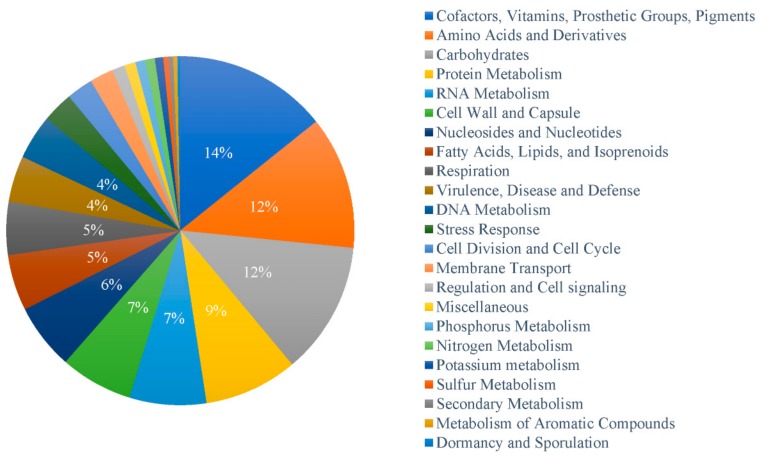
Functional distribution of genes that present in the genome of other *Dyadobacter* species that are missing in *D. tibetensis* Y620-1.

The genus *Dyadobacter* showed a conserved range of the coding density around 1.2 genes per 1 kb sequences (adjusted to 0.12 genes per 100 bp sequence, Figure 4), slightly higher than the average coding density of prokaryotic species (one gene per 1 kb of sequence) [51]. Protein coding sequences (CDS) that cannot be assigned to any known function or gene family may represent new genes [52]. We analyzed the density of new genes (genes of function unknown) in the *Dyadobacter* genomes. The results showed that the density of new genes vary greatly, ranging from 17% to 34% in *Dyadobacter* genomes (22% in average) (Figure 4). Strain *D. tibetensis* Y620-1 has the highest density of new genes of 34%, more than ten percent higher than that of the other isolates (the metagenome assembled genome was not included for its relative low completeness), and was twice that of strain *D. soli* DSM 25329 isolated from farm soil near Daejeon, South Korea [43]. It is worth noting that 771 genes with a known function present in the genome of other *Dyadobacter* species are missing in *D. tibetensis*. These genes are most related to Cofactors/Vitamins/Prosthetic Groups/Pigments (110 genes, 14%), Amino Acids and Derivatives (95 genes, 12%), Carbohydrates (95 genes, 12%), Protein Metabolism (67 genes, 9%) and RNA Metabolism (55 genes, 7%) (Figure 5).

### 3.5. Specific Functions in One-Carbon Metabolism of D. tibetensis Y620-1

**Figure 6:**
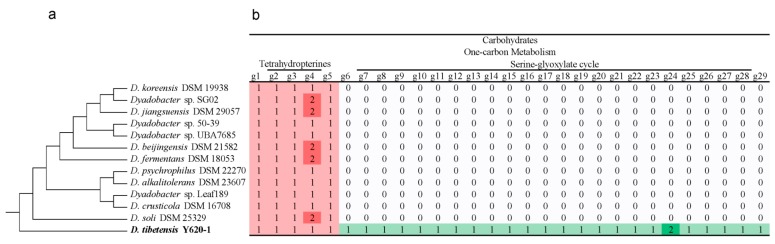
(**a**) Cladogram of *Dyadobacter* strains based on PhyloPhlAn tree; (**b**) Presence and absence of genes affiliated to the RAST category carbohydrates and subcategory one-carbon metabolism, numbers in the boxes represent copies of the related genes. Gene 1-29 represent: Formyltetrahydrofolate deformylase (EC 3.5.1.10), 5-formyltetrahydrofolate cyclo-ligase (EC 6.3.3.2), Methylenetetrahydrofolate dehydrogenase (NADP+) (EC 1.5.1.5), 5,10-methylenetetrahydrofolate reductase (EC 1.5.1.20), Methenyltetrahydrofolate cyclohydrolase (EC 3.5.4.9), Malate dehydrogenase (EC 1.1.1.37), Serine hydroxymethyltransferase (EC 2.1.2.1), Methylmalonyl-CoA mutase, small subunit (EC 5.4.99.2), Succinate dehydrogenase flavoprotein subunit (EC 1.3.99.1), Aconitate hydratase (EC 4.2.1.3), Succinate dehydrogenase iron-sulfur protein (EC 1.3.99.1), Citrate synthase (si) (EC 2.3.3.1), Propionyl-CoA carboxylase beta chain (EC 6.4.1.3). Methylmalonyl-CoA mutase (EC 5.4.99.2), 5,10-methylenetetrahydrofolate reductase (EC 1.5.1.20), Methenyltetrahydrofolate cyclohydrolase (EC 3.5.4.9), Enolase (EC 4.2.1.11), Methylcrotonyl-CoA carboxylase carboxyl transferase subunit (EC 6.4.1.4), 5-formyltetrahydrofolate cyclo-ligase (EC 6.3.3.2), Phosphoenolpyruvate carboxykinase [ATP] (EC 4.1.1.49), Putative malate dehydrogenase (EC 1.1.1.37), similar to archaeal MJ1425, Methylenetetrahydrofolate dehydrogenase (NADP+) (EC 1.5.1.5), cytosolic long-chain acyl-CoA thioester hydrolase family protein, Acetyl-CoA acetyltransferase (EC 2.3.1.9), Succinyl-CoA ligase [ADP-forming] alpha chain (EC 6.2.1.5), 3-ketoacyl-CoA thiolase (EC 2.3.1.16), low-specificity D-threonine aldolase, Succinyl-CoA ligase [ADP-forming] beta chain (EC 6.2.1.5), and Glycerate kinase (EC 2.7.1.31).

We analyzed the strain specific function of *D. tibetensis* Y620-1 and 30 genes assigned in one-carbon metabolism were detected. This was substantially higher than the other strains, which typically only contained 5–7. These genes were further divided into two subsystems: one-carbon metabolism by tetrahydropterines and serine-glyoxylate cycle (Figure 6). In the 12 reference strains, all the one-carbon metabolism related genes belonged to the subsystem tetrahydropterines. Genes related to tetrahydropterines were also present in strain *D. tibetensis* Y620-1. However, 25 genes in the subsystem serine-glyoxylate cycle that were presented in strain *D. tibetensis* Y620-1 were absent from the 12 reference strains (Figure 6).

## 4. Discussion

The 13 *Dyadobacter* genomes showed high genetic diversity in genome size, GC content, rRNA operon copy number and the number of cold shock protein genes. These features may enable them to colonize in diverse habitats such as plants, soils, freshwater, seawater, subterranean sediment sample and desert sand [27]. However, the strain *D. tibetensis* Y620-1 isolated from a deep ice core of Yuzhufeng glacier is located in the basal position of the *Dyadobacter* phylogenomic tree and separated from other freshwater isolated strains and the psychrophilic strain *D. psychrophilus* DSM 22270. Thus, strain *D. tibetensis* Y620-1 may represent a highly glacier-adapted species. NMDS analysis of gene families reveals a clear separation of *D. tibetensis* Y620-1 from the mesophilic strains, suggesting glacial environment adaptation. The well-characterized psychrophilic strain *D. psychrophilus* DSM 22270 is also clearly separated from the mesophilic strains. However, the strain *D. tibetensis* Y620-1 and *D. psychrophilus* DSM 22270 are located far away from each other (the strain *D. tibetensis* Y620-1 in the bottom-left of the plot while the strain *D. psychrophilus* DSM 22270 in the upper-right of the plot). The separation of the two cold adapted strains in the NMDS plot may reveal different functions of the two strains. Strain Y620-1 was isolated from a glacier ice core, where the primary productivity is much lower than that of soil. Thus, the ability in carbon metabolism may differ between these two psychrophiles.

A limited difference was detected at the category and subcategory levels. However, at the subsystem level, presence and absence of genes related to one-carbon metabolism could clearly differentiate *D. tibetensis* Y620-1 from the other 12 reference strains. Genes related to serine-glyoxylate cycle present in *D. tibetensis* Y620-1 are not identified from any of the 12 reference strains. One-carbon compounds can be generated from various renewable sources, such as digestion of organic matter [53]. The serine-glyoxylate cycle is unique since it is the only naturally evolved oxygen-insensitive pathway that can synthesize acetyl-CoA (the two-carbon building block) from multiple groups of one-carbon compounds without carbon loss [54]. In the oligotrophic glacial environment, one of the survival challenges is to obtain metabolic substrates [55]. One-carbon compounds may support microbial communities in the cold and oligotrophic environment [56]. The presence of genes relate to serine-glyoxylate cycle may enable the strain *D. tibetensis* Y620-1 to utilize simply formed and newly produced carbon sources, e.g., decomposed microbial residues entrapped in the glacier and labile organic carbon freshly derived from photosynthetic bacteria [57,58,59,60]. The carbon and energy sources in the veins of the ice core were estimated to be able to maintain the bacterial population for thousands of years [8]. Oligotrophic lifestyle could also be revealed by the lower copy number of rRNA operon in *D. tibetensis* Y620-1 [49]. All genes required for serine-glyoxylate cycle [54] are found and are specific to the glacier isolated strain *D. tibetensis* Y620-1, suggesting the utilization of one-carbon may be one of the strategies for adaptation to the oligotrophic condition in the glacier environments.

Low-temperature habitats are hot spots of microbial diversity and evolution. These environments may harbor microorganisms that process novel metabolic functions [61]. Our results showed that *D. tibetensis* Y620-1 had the highest density of novel genes compared with other genomes. The basal placement of *D. tibetensis* Y620-1 in the phylogenomic tree suggests that these new genes and functions could be ancient origin. This is supported by the view that distribution of bacteria may not result from widespread contemporary dispersal but is an ancient evolutionary legacy, as revealed by the evolutional analysis of cold desert cyanobacteria and thermal traits of Streptomyces sister-taxa [62,63].

## Figures and Tables

**Table 1:** Genomic and phenotypic characteristics of the 13 *Dyadobacter* strains with sequenced genomes.

Strain	Assembly No.	Isolation Sources	Completeness	Contamination	GC	Size (Mbp)	CDS	CRISPRs	rRNAs	tRNAs	CspA	CspG	New Gene Dendity	Coding Density
*D. alkalitolerans* DSM 23607	GCA_000428845.1	Desert sand	100.00	0.00	45.67	6.29	5496	0	3	35	3	1	0.24	0.11
*D. beijingensis* DSM 21582	GCA_000382205.1	Soil	99.69	0.30	52.08	7.38	6030	0	6	40	4	1	0.23	0.12
*D. crusticola* DSM 16708	GCA_000701505.1	Soil	100.00	0.00	46.73	6.07	5141	0	3	40	2	1	0.20	0.12
*D. fermentans* DSM 18053	GCA_000023125.1	Plant	99.70	0.30	51.54	6.97	5853	0	12	43	2	1	0.22	0.12
*D. jiangsuensis* DSM 29057	GCA_003014695.1	Soil	100.00	0.60	50.26	8.27	6854	0	2	38	2	1	0.19	0.12
*D. koreensis* DSM 19938	GCA_900108855.1	Fresh water	99.70	0.89	41.26	7.34	6140	0	7	40	1	1	0.19	0.12
*D. psychrophilus* DSM 22270	GCA_900167945.1	Soil	99.70	0.30	45.05	6.74	5722	0	4	34	2	1	0.19	0.12
*D. soli* DSM 25329	GCA_900101885.1	Soil	99.70	0.00	50.47	8.74	7339	0	6	40	1	1	0.17	0.12
*D. tibetensis* Y620-1	GCA_000566685.1	Ice core	99.70	0.30	43.45	5.31	4275	6	3	37	5	2	0.34	0.12
*Dyadobacter* sp. 50-39	GCA_001898145.1	Bioreactor	99.70	0.60	50.24	7.72	6563	5	2	40	4	1	0.20	0.12
*Dyadobacter* sp. Leaf189	GCA_001424405.1	Leaf	99.70	0.60	47.00	6.07	5141	0	3	40	3	1	0.24	0.12
*Dyadobacter* sp. SG02	GCA_900109045.1	Root	99.70	0.74	50.23	8.48	7043	0	2	38	6	1	0.21	0.12
*Dyadobacter* sp. UBA7685	GCA_002482895.1	Water	97.02	0.00	50.58	5.18	4436	0	0	30	2	1	0.27	0.12

## Supplementary Materials

Supplementary materials can be found at https://www.mdpi.com/2076-2607/7/7/211/s1.

## References

[1] Grinsted A. (2013). An estimate of global glacier volume. Cryosphere.

[2] Anesio A.M., Laybourn-Parry J. (2012). Glaciers and ice sheets as a biome. Trends Ecol. Evol..

[3] Hodson A., Anesio A.M., Tranter M., Fountain A., Osborn M., Priscu J., Laybourn-Parry J., Sattler B. (2008). Glacial ecosystems. Ecol. Monogr..

[4] Larose C., Dommergue A., Vogel T.M. (2013). Microbial nitrogen cycling in Arctic snowpacks. Environ. Res. Lett..

[5] Maccario L., Sanguino L., Vogel T.M., Larose C. (2015). Snow and ice ecosystems: Not so extreme. Res. Microbiol..

[6] Laybourn-Parry J. (2009). No Place Too Cold. Science.

[7] Christner B.C., Mosley-Thompson E., Thompson L.G., Zagorodnov V., Sandman K., Reeve J.N. (2000). Recovery and identification of viable bacteria immured in glacial ice. Icarus.

[8] Price P.B. (2000). A habitat for psychrophiles in deep Antarctic ice. Proc. Natl. Acad. Sci. USA.

[9] Frasson D., Udovicic M., Frey B., Lapanje A., Zhang D.C., Margesin R., Sievers M. (2015). *Glaciimonas alpina* sp. nov. isolated from alpine glaciers and reclassification of *Glaciimonas immobilis* Cr9-12 as the type strain of *Glaciimonas alpina* sp. nov. Int. J. Syst. Evol. Microbiol..

[10] Margesin R., Schumann P., Zhang D.C., Redzic M., Zhou Y.G., Liu H.C., Schinner F. (2012). Arthrobacter cryoconiti sp nov., a psychrophilic bacterium isolated from alpine glacier cryoconite. Int. J. Syst. Evol. Microbiol..

[11] Margesin R., Sproer C., Schumann P., Schinner F. (2003). *Pedobacter cryoconitis* sp. nov., a facultative psychrophile from alpine glacier cryoconite. Int. J. Syst. Evol. Microbiol..

[12] Liu Q., Liu H.C., Wen Y., Zhou Y.G., Xin Y.H. (2012). *Cryobacterium flavum* sp. nov. and *Cryobacterium luteum* sp. nov., isolated from glacier ice. Int. J. Syst. Evol. Microbiol..

[13] Pal M., Kumari M., Kiran S., Salwan R., Mayilraj S., Chhibber S., Gulati A. (2018). *Chryseobacterium glaciei* sp. nov., isolated from the surface of a glacier in the Indian trans-Himalayas. Int. J. Syst. Evol. Microbiol..

[14] Dong K., Liu H.C., Zhang J.L., Zhou Y.G., Xin Y.H. (2012). *Flavobacterium xueshanense* sp. nov. and *Flavobacterium urumqiense* sp. nov., two psychrophilic bacteria isolated from glacier ice. Int. J. Syst. Evol. Microbiol..

[15] Shen L., Liu Y.Q., Wang N.L., Yao T.D., Jiao N.Z., Liu H.C., Zhou Y.G., Xu B.Q., Liu X.B. (2013). *Massilia yuzhufengensis* sp. nov., isolated from an ice core. Int. J. Syst. Evol. Microbiol..

[16] Shen L., Liu Y.Q., Yao T.D., Wang N.L., Xu B.Q., Jiao N.Z., Liu H.C., Zhou Y.G., Liu X.B., Wang Y.N. (2013). *Dyadobacter tibetensis* sp. nov., isolated from glacial ice core. Int. J. Syst. Evol. Microbiol..

[17] Bajerski F., Ganzert L., Mangelsdorf K., Lipski A., Busse H.J., Padur L., Wagner D. (2013). *Herbaspirillum psychrotolerans* sp. nov., a member of the family Oxalobacteraceae from a glacier forefield. Int. J. Syst. Evol. Microbiol..

[18] Zhang Y.M., Jiang F., Chang X.L., Qiu X., Ren L.Z., Qu Z.H., Deng S.S., Da X.Y., Fang C.X., Peng F. (2016). *Flavobacterium collinsense* sp. nov., isolated from a till sample of an Antarctic glacier. Int. J. Syst. Evol. Microbiol..

[19] Qiu X., Qu Z.H., Jiang F., Ren L.Z., Chang X.L., Kan W.J., Fang C.X., Peng F. (2014). *Pedobacter huanghensis* sp. nov. and *Pedobacter glacialis* sp. nov., isolated from Arctic glacier foreland. Int. J. Syst. Evol. Microbiol..

[20] Srinivas T.N.R., Manasa P., Begum Z., Sunil B., Sailaja B., Singh S.K., Prasad S., Shivaji S. (2013). *Iodobacter arcticus* sp. nov., a psychrotolerant bacterium isolated from meltwater stream sediment of an Arctic glacier. Int. J. Syst. Evol. Microbiol..

[21] Zeng Y.X., Yu Y., Liu Y., Li H.R. (2016). *Psychrobacter glaciei* sp. nov., isolated from the ice core of an Arctic glacier. Int. J. Syst. Evol. Microbiol..

[22] Price P.B. (2007). Microbial life in glacial ice and implications for a cold origin of life. FEMS Microbiol. Ecol..

[23] Yadav A.N., Sachan S.G., Verma P., Kaushik R., Saxena A.K. (2016). Cold active hydrolytic enzymes production by psychrotrophic Bacilli isolated from three sub-glacial lakes of NW Indian Himalayas. J. Basic. Microbiol..

[24] Rodrigues D.F., Tiedje J.M. (2008). Coping with our cold planet. Appl. Environ. Microbiol..

[25] De Maayer P., Anderson D., Cary C., Cowan D.A. (2014). Some like it cold: Understanding the survival strategies of psychrophiles. EMBO Rep..

[26] Chelius M.K., Triplett E.W. (2000). *Dyadobacter fermentans* gen. nov., sp. nov., a novel Gram-negative bacterium isolated from surface-sterilized Zea mays stems. Int. J. Syst. Evol. Microbiol..

[27] Gao J.L., Sun P.B., Wang X.M., Qiu T.L., Lv F.Y., Yuan M., Yang M.M., Sun J.G. (2016). *Dyadobacter endophyticus* sp. nov., an endophytic bacterium isolated from maize root. Int. J. Syst. Evol. Microbiol..

[28] Liu Y.Q., Hu A.Y., Shen L., Yao T.D., Jiao N.Z., Wang N.L., Xu B.Q. (2014). Draft genome sequence of *Dyadobacter tibetensis* type strain (Y620-1) isolated from glacial ice. Stand. Genom. Sci..

[29] Parks D.H., Imelfort M., Skennerton C.T., Hugenholtz P., Tyson G.W. (2015). CheckM: Assessing the quality of microbial genomes recovered from isolates, single cells, and metagenomes. Genome Res..

[30] CompareM. https://github.com/dparks1134/CompareM.

[31] ANI Calculator. http://enve-omics.ce.gatech.edu/ani/.

[32] Overbeek R., Olson R., Pusch G.D., Olsen G.J., Davis J.J., Disz T., Edwards R.A., Gerdes S., Parrello B., Shukla M. (2014). The SEED and the Rapid Annotation of microbial genomes using Subsystems Technology (RAST). Nucleic Acids Res..

[33] Torsten S. (2014). Prokka: Rapid prokaryotic genome annotation. Bioinformatics.

[34] Yarza P., Richter M., Peplies J., Euzeby J., Amann R., Schleifer K.H., Ludwig W., Glöckner F.O., Rosselló-Móra R. (2008). The All-Species Living Tree project: A 16S rRNA-based phylogenetic tree of all sequenced type strains. Syst. Appl. Microbiol..

[35] Yang Z.H. (2006). Computational Molecular Evolution.

[36] Segata N., Bornigen D., Morgan X.C., Huttenhower C. (2013). PhyloPhlAn is a new method for improved phylogenetic and taxonomic placement of microbes. Nat. Commun..

[37] Tamura K., Peterson D., Peterson N., Stecher G., Nei M., Kumar S. (2011). MEGA5: Molecular evolutionary genetics analysis using maximum likelihood, evolutionary distance, and maximum parsimony methods. Mol. Biol. Evol..

[38] Ronquist F., Teslenko M., van der Mark P., Ayres D.L., Darling A., Hohna S., Larget B., Liu L., Suchard M.A., Huelsenbeck J.P. (2012). MrBayes 3.2: Efficient Bayesian phylogenetic inference and model choice across a large model space. Syst. Biol..

[39] Parks D.H., Rinke C., Chuvochina M., Chaumeil P.A., Woodcroft B.J., Evans P.N., Hugenholtz P., Tyson G.W. (2017). Recovery of nearly 8000 metagenome-assembled genomes substantially expands the tree of life. Nat. Microbiol..

[40] FastOrtho Software (--pv_cutoff 1-e5 --pi_cutoff 70 --pmatch_cutoff 70). http://enews.patricbrc.org/fastortho/.

[41] Dixon P. (2003). VEGAN, a package of R functions for community ecology. J. Veg. Sci..

[42] Wang L., Chen L., Ling O., Li C.C., Tao Y., Wang M. (2015). *Dyadobacter jiangsuensis* sp. nov., a methyl red degrading bacterium isolated from a dye-manufacturing factory. Int. J. Syst. Evol. Microbiol..

[43] Lee M., Woo S.G., Park J., Yoo S.A. (2010). *Dyadobacter soli* sp. nov., a starch-degrading bacterium isolated from farm soil. Int. J. Syst. Evol. Microbiol..

[44] Zhang D.C., Liu H.C., Xin Y.H., Zhou Y.G., Schinner F., Margesin R. (2010). *Dyadobacter psychrophilus* sp. nov., a psychrophilic bacterium isolated from soil. Int. J. Syst. Evol. Microbiol..

[45] Dong Z., Guo X.Y., Zhang X.X., Qiu F.B., Sun L., Gong H.L., Zhang F.Y. (2007). *Dyadobacter beijingensis* sp. nov, isolated from the rhizosphere of turf grasses in China. Int. J. Syst. Evol. Microbiol..

[46] Tang Y.L., Dai J., Zhang L., Mo Z.Y., Wang Y., Li Y.W., Ji S.M., Fang C.X., Zheng C.Y. (2009). *Dyadobacter alkalitolerans* sp. nov., isolated from desert sand. Int. J. Syst. Evol. Microbiol..

[47] Reddy G.S.N., Garcia-Pichel F. (2005). *Dyadobacter crusticola* sp. nov., from biological soil crusts in the Colorado Plateau, USA, and an emended description of the genus *Dyadobacter* Chelius and Triplett 2000. Int. J. Syst. Evol. Microbiol..

[48] Baik K.S., Kim M.S., Kim E.M., Kim H.R., Seong C.N. (2007). *Dyadobacter koreensis* sp. nov., isolated from fresh water. Int. J. Syst. Evol. Microbiol..

[49] Rawat S.R., Bromberg Y., Häggblom M.M., Männistö M.K. (2012). Comparative genomic and physiological analysis provides insights into the role of Acidobacteria in organic carbon utilization in Arctic tundra soils. FEMS Microbiol. Ecol..

[50] Konstantinidis K.T., Tiedje J.M. (2005). Genomic insights that advance the species definition for prokaryotes. Proc. Natl. Acad. Sci. USA.

[51] Konstantinidis K.T., Tiedje J.M. (2004). Trends between gene content and genome size in prokaryotic species with larger genomes. Proc. Natl. Acad. Sci. USA.

[52] Mukherjee S., Seshadri R., Varghese N.J., Eloe-Fadrosh E.A., Meier-Kolthoff J.P., Goker M., Coates R.C., Hadjithomas M., Pavlopoulos G.A., Paez-Espino D. (2017). 1003 reference genomes of bacterial and archaeal isolates expand coverage of the tree of life. Nat. Biotechnol..

[53] Sawatdeenarunat C., Nguyen D., Surendra K.C., Shrestha S., Rajendran K., Oechsner H., Xie L., Khanal S.K. (2016). Anaerobic biorefinery: Current status, challenges, and opportunities. Bioresour. Technol..

[54] Smejkalova H., Erb T.J., Fuchs G. (2010). Methanol assimilation in Methylobacterium extorquens AM1: Demonstration of all enzymes and their regulation. PLoS ONE.

[55] Murakami T., Segawa T., Bodington D., Dial R., Takeuchi N., Kohshima S., Hongoh Y. (2015). Census of bacterial microbiota associated with the glacier ice worm Mesenchytraeus solifugus. FEMS Microbiol. Ecol..

[56] Ji M., Greening C., Vanwonterghem I., Carere C.R., Bay S.K., Steen J.A., Montgomery K., Lines T., Beardall J., van Dorst J. (2017). Atmospheric trace gases support primary production in Antarctic desert surface soil. Nature.

[57] Singh P., Singh S.M., Dhakephalkar P. (2014). Diversity, cold active enzymes and adaptation strategies of bacteria inhabiting glacier cryoconite holes of High Arctic. Extremophiles.

[58] McCrimmon D.O., Bizimis M., Holland A., Ziolkowski L.A. (2018). Supraglacial microbes use young carbon and not aged cryoconite carbon. Org. Geochem..

[59] Jungblut A.D., Mueller D., Vincent W.F. (2017). Arctic Ice Shelves and Ice Islands.

[60] Smith G.J., Foster R.A., McKnight D.M., Lisle J.T., Littmann S., Kuypers M.M.M., Foreman C.M. (2017). Microbial formation of labile organic carbon in Antarctic glacial environments. Nat. Geosci..

[61] Anesio A.M., Bellas C.M. (2011). Are low temperature habitats hot spots of microbial evolution driven by viruses?. Trends Microbiol..

[62] Bahl J., Lau M.C., Smith G.J., Vijaykrishna D., Cary S.C., Lacap D.C., Lee C.K., Papke R.T., Warren-Rhodes K.A., Wong F.K. (2011). Ancient origins determine global biogeography of hot and cold desert cyanobacteria. Nat. Commun..

[63] Choudoir M.J., Buckley D.H. (2018). Phylogenetic conservatism of thermal traits explains dispersal limitation and genomic differentiation of *Streptomyces* sister-taxa. ISME J..

